# What Drives Them to Drive?—Parents' Reasons for Choosing the Car to Take Their Children to School

**DOI:** 10.3389/fpsyg.2017.01970

**Published:** 2017-11-08

**Authors:** Jessica Westman, Margareta Friman, Lars E. Olsson

**Affiliations:** Service Research Center (CTF) and Samot VINN Excellence Center, Karlstad University, Karlstad, Sweden

**Keywords:** school travel, stated reasons, car choice, parental decision, children and adolescents

## Abstract

Children's school journeys have changed vastly during recent decades: More children are being driven to school in private cars instead of walking and cycling, with many who are entitled to a free school bus service still being driven. Earlier research into travel mode choice has often investigated how urban form impacts upon mode choice regarding school journeys—in particular how urban form hinders or enables the use of the active mode. This paper quantitatively explores parents' stated reasons for choosing the car and the relationship between these reasons and the decision to use the car to take their children to school. We additionally investigate the relationship between sociodemographic factors, distance, and both the stated reasons and the actual mode decision. A sample of 245 parents (194 women) of school children aged 10–15 in the County of Värmland in Sweden were included in the study. The results of PLS-SEM show that the factor Social convenience has a direct relationship with the frequency of car use indicating that the wish to accompany the child and the convenience of car impacts on car choice. If the child is not allowed to travel independently, the parents choose the car to take him/her to school. Sociodemographic factors had a direct relationship with the stated reasons, whereby parents with a higher level of education valued safety/security less. Quite surprisingly, distance (i.e., environmental factor) did not affect car use, indicating that parents drive their children to school regardless of distance. By isolating the particular reasons for choosing the car, this paper focuses on a potentially important missing piece as regards finding out what motivates the increasing car usage in children's school journeys. An increased knowledge of what motivates the decision to take children by car is important for effective policies aimed at changing parents' inclination toward choosing the car.

## Introduction

Building upon previous research, the aim of this study is to investigate the reasons parents state for choosing the car to take their children to school. A contribution with this study is to gather earlier findings on parental motives for car use and analyze them simultaneously in order to explain relationships between a number of identified factors and frequency of car use. Specifically, we explore and investigate factors related to environmental and sociodemographic details, but also include parental stated reasons for choosing the car. By focusing on the stated reasons for the specific car choice, we gain a more nuanced understanding of factors underlying decisions relating to car use.

During previous decades, children's school journey patterns have been changing; they are traveling both longer distances and more frequently by car (Faulkner et al., 2010; Andersson et al., 2012). The reduction in the use of independent (i.e., travels made without adult supervision) and active travel seems to be a general issue in countries in the developed world where the preference for the car surpasses other alternatives (McDonald et al., 2011; Mackett, 2013). However, this is a trend also notable in some low- and middle-income countries, e.g., Vietnam (Mammen et al., 2012; Trang et al., 2012; Mackett, 2013). The increasing use of the car far exceeds the increase in distance; a large number of parents who recognize that the distance involved is close enough for their children to use an active mode (walk or cycle) still drive these children to school (Lee et al., 2013; He and Giuliano, 2017). Additionally, many children in Europe and the US, who are entitled to free school bus service, are also being driven to school by car (Ewing et al., 2007). This negative trend has intrigued researchers to find out what either hinders or motivates the use of an active mode by often investigating environmental factors (e.g., traffic and urban form) and sociodemographic factors (e.g., age, gender, income, and occupational status) (Davison et al., 2008; McDonald et al., 2014). Common practice has been to investigate these factors and compare them to escorted and unescorted journeys, often omitting, however, the impact of parents' personal reasons for their choice of travel mode (Susilo and Liu, 2016; Mah et al., 2017). This limits the broader literature on children's daily travel, since research rarely regard the escort decision and the travel mode decision to have been underpinned by different parental decision-making processes (Mammen et al., 2012). By combining sociodemographic and environmental factors with parents' stated reasons, we may gain a broader knowledge of parental travel mode choice.

In reviewing the broader literature on children and travel the increasing use of the car for school journeys stems from issues relating to the built environment. Research show that long distances from home to school and congestion constrain active and/or independent travel (without adult supervision) in favor of the car (Ferdinand et al., 2012; Gustat et al., 2014a,b; Larsen et al., 2015). The difference between motorized and non-motorized transportation seems to lie within a limit of 2 km (Kelly and Fu, 2014), with parents assessing the suitability of the physical environment before deciding on travel mode (Johansson, 2006; Race et al., 2017). However, many parents drive their children even though the environment meets walkable requirements (e.g., proximity, the presence of cycle lanes, the location of speed bumps, sidewalk coverage), indicating that additional factors underlie decisions to take the car (Mehdizadeh et al., 2016; Mah et al., 2017).

On the societal level, the increasing number of car journeys among Scandinavian children has been explained by changes in mothers' employment rates, which have increased household time constraints and car use (Panter et al., 2013). The children of well-educated parents, from households with a higher income, a higher level of car-ownership, and more than one child, are more likely to travel to school by car (McMillan, 2003; Chillón et al., 2014; Mehdizadeh et al., 2016). The characteristics of the child impact upon mode choice whereby older children (>11) use the car less frequently and are more likely to travel actively than younger children are (McDonald et al., 2011). Additionally, girls are more often being escorted by their parents in cars than boys (McMillan, 2006).

Research into motives for children's mode choice show that attitudes (here referred to as the degree to which a parent holds a favorable or an unfavorable evaluation of a specific travel mode such as the car) influence travel mode choice more so than the attributes of the built environment (McMillan, 2007). Just as in research on adult travel behavior (Steg, 2005; Gärling and Schuitema, 2007; Ettema et al., 2012), convenience of car is highlighted. Dropping children off on the way to work (trip-chaining) makes chaotic mornings feel less stressful—especially when several children from the same household are involved (Ahlport, 2008; Eyler et al., 2013; Scheiner and Holz-Rau, 2017). Parents also claim that driving enables them to spend time with their child (Barker, 2009). Parents who regularly choose the car seem to have different thoughts regarding what constitutes a close enough distance to enable walking or cycling to school in comparison with parents who choose active travel for their children—even though they share the same distance between home and school (Lee et al., 2013). Distance between home and school may influence how parents value their child's ability to safely navigate traffic, handle social interactions, and avoid potentially dangerous situations, in turn influencing the decision to take (or not to take) the car (Stewart et al., 2012). Parents who regularly use the car perceive the built environment to be more hazardous than parents who allow their children to walk or cycle (Johansson, 2006). Thus, parents appear to value safety differently even though they share the same objective environment, indicating that safety is not merely a matter of environmental elements but is subjectively evaluated (Hume et al., 2009; Seraj et al., 2012; Lee et al., 2013; Mah et al., 2017). Steg (2005) argues that affective motives underlie car choice regarding adults' trips. These motives entail feelings of arousal and sensations but that there is also a sheer enjoyment of driving which provides a sense of independence, which could also apply when choosing mode for their children. However, there is no clear and unanimous consensus as to what these parental reasons are or entail (Lee et al., 2017) and this paper aims to gain a better understanding of what factors that drive parents to drive their children to school. Specifically, we investigate the relationship between environmental and sociodemographic factors and car choice, but also address parents' stated reasons that tap into sense of security, convenience, and the opportunity to accompany the child. As described earlier, previous research into children and travel indicates that the travel mode decision is partly based on factors related to practicalities, safety concerns and the wish for social interaction (McMillan, 2007; McDonald et al., 2016; Ahern et al., 2017) and this study explore this further. This is important because the current increase in children's car travels is unsustainable from both an environmental and health point of view, primarily owing to that car travel produces greenhouse gases contributing to global climate change as well as foster less physical activity among schoolchildren.

## Materials and methods

### Setting

To achieve sufficient variation in both travel distance and sociodemographic factors, five different schools in Värmland County (~273,000 inhabitants), in southwest Sweden, were selected to participate in the study. Two of the schools were in Arvika Municipality (with a population of ~26,000, geographic area 1,659 km^2^), two in Torsby Municipality (with a population of ~12,700, geographic area 4,187 km^2^), and one in Karlstad Municipality (with a population of ~84,000, geographic area 1,167 km^2^). The schools varied in their numbers of students (Torsby having 400 and 35, Arvika 420 and 230, and Karlstad 450) and in their distances between students' homes and school (the median distance in Torsby being 4.5 km, Arvika 2 km, and Karlstad 2.5 km). Data collection took place between December 2012 and March 2013. The weather conditions were normal for the season and the outside temperature varied between +1 and −20°C (34 and −4°F), with the depth of snow varying between a few centimeters and a few decimeters.

## Participants

In one of our earlier studies (i.e., Westman et al., 2016) of children's travel experiences, 345 school children (aged 10–15) participated. In the present study, we approached these children's parents (or guardians) and the parents of the children who had been unable to participate (e.g., due to vacation or sick leave). A letter was sent to 425 homes asking one parent in each household if he or she was willing to participate, via either a web-based survey or a written questionnaire sent through the post. If a parent had more than one child participating in the study, then he/she was asked to fill out one questionnaire for each child. Data were obtained for 245 parents (79% women), with about 50% of these 245 choosing to answer the questionnaire online. The total response rate was ~58% (93 respondents from Torsby, 108 from Arvika, and 44 from Karlstad). In 37% of households, the responding parent had a university degree, with a monthly household net income of ~SEK 45 k. All households had access to at least one car. These statistics are close to the county averages (Statistics Sweden, 2016).

## Measures and analysis

In this paper, a range of measurements was used to capture why parents choose the car as their children's mode of traveling to school. Distance (i.e., environmental factor) was captured by asking the parents how many kilometers their homes were from school. The distances were 0–1 km (*n* = 61), 1–2 km (*n* = 34), 2–3 km (*n* = 18), 3–4 km (*N* = 27), ≥5 km (*n* = 103) (missing values, *n* = 3). To investigate how parental and child sociodemographics impact upon the choice of taking the car, we asked the parents to provide information about their children's age and gender as well as background questions (i.e., parental gender, academic degree, income, car ownership, and occupational status). Based on earlier research into parental travel mode choice (Johansson, 2006; McMillan, 2007; Stewart et al., 2012; McDonald et al., 2016; Ahern et al., 2017), a number of possible reasons for car choice were given. Thus, the parents stated their reasons for choosing the car on a five-point rating scale, ranging from *disagree completely* (1) to *agree completely* (5). The alternatives were: opportunity to spend time with a parent, concerns over traffic dangers, concerns over stranger danger, worries about child being bullied by other children, conveniently going that way, accompaniment by adult (Kyttä, 2004). The parents marked how many days during the school week (ranging from 0 days a week to 5) the car was used to school (0 days = 116, 1 day = 43, 2 days = 18, 3 days = 10, 4 days = 5, and 5 days = 40), (missing values, *n* = 13), which was used as the dependent variable in the analyses. Finally, to report independent travel, the parents marked whether or not their children were allowed to travel alone to school by answering a *yes or no* question (1 = yes, 2 = no).

We analyzed the data using three analytical techniques, i.e., factor analysis, partial least square structural equation modeling (PLS-SEM), and correlation analysis. We built the independent latent variables using exploratory factor analysis (Henson and Roberts, 2006) by means of extracting the principal components. The reliability and validity of the measures were then analyzed using partial least squares (PLS-SEM). PLS-SEM is a robust method that allows the analysis of small-sample data with non-normal distributions (Hair et al., 2017). Direct effects, indirect effects, and total effects can be estimated, along with the psychometric properties of the measurement model and the parameters of the structural model. PLS-SEM is a valuable tool for exploratory research (Hair et al., 2014), and thus applicable to our material.

## Results

### Factor analysis and relationships between main variables

We first examined the factorability of the six stated reasons. The Kaiser-Meyer-Olkin measure of sampling adequacy was 0.70, which is above the commonly recommended value of 0.6, while Bartlett's test of sphericity was significant [$\chi_{\left(15\right)}^{2}$ = 295.56, *p* < 0.001]. Principal Component Analysis yielded two factors with eigenvalues > 1.0, jointly accounting for 63% of the variance. The six items correlated with at least 0.3 with one factor. Substantial loading was observed for the first rotated factor measuring stranger danger, traffic danger, concerns about being bullied by other children; we identified this factor as *Safety/Security*. The second factor was identified as *Social convenience* and measured accompaniment by an adult, conveniently going that way, and the opportunity to spend time with an adult (see Table 1), these factors were used as latent variables in the following analyses. In Table 2, relationships between the main variables are described. As can be seen, there is a significant positive relationship between Safety/security and frequency of car travel as well as between Social convenience and frequency of car travel. The child's age was negatively correlated with Safety/Security as was Degree of education on Safety/security. Details of all correlations can be found in Table 2.

**Table 1:** Varimax rotated loadings from principal component analysis.

**Item**	**Factor 1 safety/security**	**Factor 2 social convenience**
Stranger danger	0.792	
Traffic danger	0.763	
Worry of bullying	0.807	
Accompaniment by parent/adult		0.808
Opportunity to spend time with parent/adult		0.683
Conveniently going that way		0.784

**Table 2:** Correlations between main variables.

	***M***	***SD***	**1**.	**2**.	**3**	**4**	**5**	**6**	**7**	**8**	**9**	**10**
1. Child gender (girls = 2, boys = 1)	1.53	0.50										
2. Child age	12.29	1.75	−0.28									
3. Parent gender (women = 194, men = 51)	1.78	0.41	0.16	0.03								
4. Education (1–4)	2.56	0.95	0.11	−0.01	0.04							
5. Income (1–6)	2.71	1.06	0.19	0.05	0.04	0.26^*^						
6. Independent travel (Yes = 1, No = 2)	1.30	0.46	0.21	−0.01	−0.03	−0.01	0.03					
7. Social convenience (1–5)	2.19	1.32	−0.00	0.01	−0.07	−0.04	−0.02	0.29^*^				
8. Safety/Security (1–5)	2.21	1.23	0.02	−0.22^*^	−0.02	−0.20^*^	−0.04	0.20^*^	0.27^*^			.
9. Distance (km)	3.32	1.69	−0.03	0.21^*^	−0.02	−0.01	−0.02	0.40^*^	0.03	−0.02		
10.Car (0–5 days a week)	1.42	1.88	0.09	0.08	−0.07	0.01	0.00	0.44^*^	0.63^*^	25^*^	0.09	

* *p < 0.005*.

### Overview of PLS-SEM

#### Validity and reliability test

Requirements for correct estimates of the effects of the latent variables (safety/security and social convenience) include reliability, convergent validity, and discriminant validity being acceptable. Initial analyses of convergent validity (to what extent the indicator variable positively correlates with alternative measures of the same construct) revealed that the items *concern about being bullied* and *the opportunity to spend time with an adult* did not reach satisfactory levels (should be >0.70); thus, these items were deleted from the latent variables, see Table 3.

**Table 3:** Results from measurement model estimation (outer loadings, composite reliability, and AVE for the latent factors Safety/Security and Social convenience).

	**Manifest variable**	**Outer loadings**	**Composite reliability**	**AVE**
Safety/security			0.85	0.75
	Traffic danger	0.89		
	Stranger danger	0.84		
Social convenience			0.83	0.71
	Accompaniment adult	0.81		
	Conveniently going that way	0.87		

The latent variables show themselves to be robust in respect of their internal consistency reliability, as indexed by composite reliability (CR). The outer loadings, which represent the loadings of the reflective manifest variables (i.e., *traffic danger, stranger danger*, and *conveniently going that way, accompaniment by an adult*), with their respective latent variable (safety/security and social convenience), assess convergent validity and exceed the recommended value of 0.7 (Roldán and Sánchez-Franco, 2012).

The average variance extracted (AVE) for each measure also exceeds the recommended value of 0.5 (Fornell and Larcker, 1981). The reliability and validity of the measure scale are given in Table 3.

The Heterotrait-monotrait ratio of correlations (HTMT) measures the extent to which a construct positively correlates with alternative measures of the same construct by extracting the factor and cross loadings of the indicator items into their respective latent construct (Hair et al., 2017), which can additionally serve as the basis of the statistical discriminant validity test. The HTMT in our model reaches satisfactory levels of 0.46, well below the suggested upper threshold of 0.85 (Henseler et al., 2015). Given that the reliability, convergent validity, and discriminant validity are satisfactory, estimations may be made of the direct, indirect, and total effects of the exogenous variables.

#### Results of model estimates

After confirming that the construct measures were valid and reliable, our next step was to evaluate a structural model. We are interested in exploring and determining the extent to which parents' stated reasons, sociodemographic factors, children's travel independence, and distance all account for car use during school journeys. More specifically, the model tests the effect of both social convenience and safety/security on car use. It further tests the effects of independent travel on car use. (It may still be the case that children are allowed to actively travel to school with friends, parents, or by school bus). It also tests the effects of sociodemographics and distance on car use but it also tests the effects of sociodemographic and environmental factors on safety/security and social convenience. We assessed the significance of the path model relationships among the constructs by using a bootstrapping procedure with a resample of 5,000. The standardized root mean square residual (SRMR) (Henseler et al., 2015) was used to assess the approximate model fit. The calculated value of 0.06 is below the cut-off value of 0.08 (Hue and Bentler, 1999), indicating an acceptable fit.

**Figure 1:**
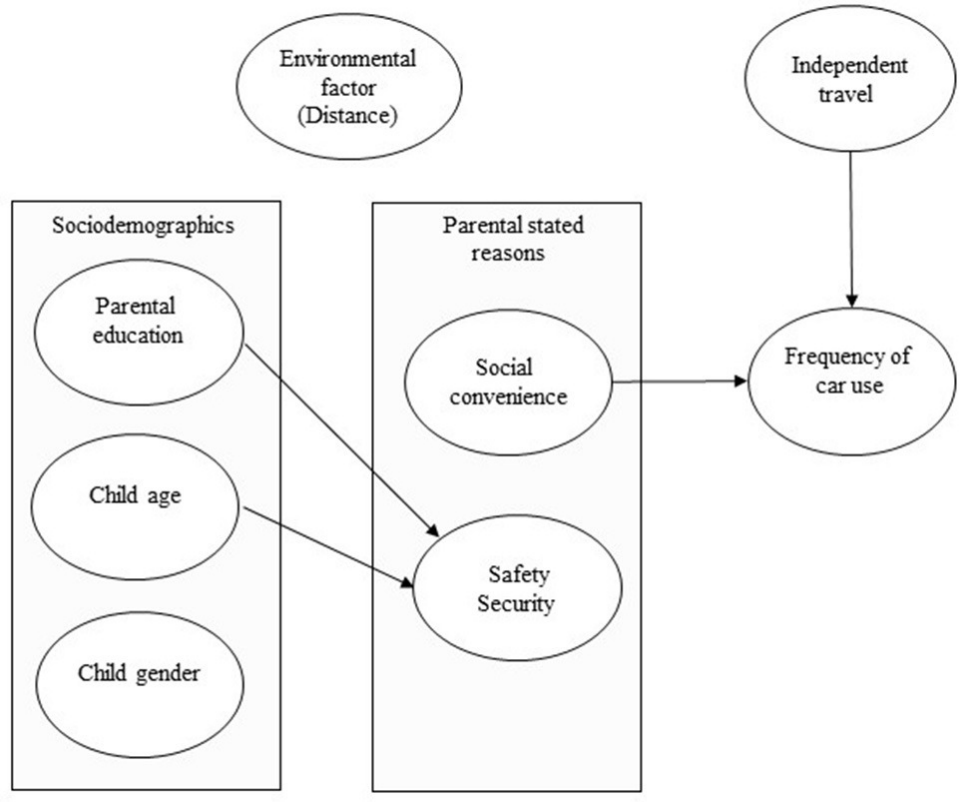
Arrows indicating significant findings.

Table 4 gives the full estimation results for car choice frequency, social convenience and safety/security (standardized estimates (β) and *p-*values for total, direct, and indirect effects). In Figure 1 the significant paths of the estimations are shown. As can be seen, social convenience was directly related to car choice; parents who highly value social convenience on the way to school were more likely to choose the car. If the child was not allowed to travel independently, then his/her parents were more likely to choose the car to take him/her to school. Sociodemographic factors were directly related to safety/security whereby parents with a higher level of education valued safety/security less. It is also important to note that no significant path was observed between distance and mode choice^1^.

**Table 4:** Standardized estimates (β), 95%, and *p* values for total, direct, and indirect effects of all paths in the PLS-SEM for Car.

	**Safety/security**		**Social convenience**		**Car use**	
	**β**	***P***	**β**	***P***	**β**	***P***
**TOTAL EFFECTS**						
Child age	−0.222	**0.002**	0.031	0.644	0.097	0.098
Child gender	0.032	0.615	0.001	0.988	0.086	0.144
Parent gender	−0.007	0.911	−0.071	0.276	−0.067	0.277
Education	−0.198	**0.002**	−0.047	0.492	−0.029	0.632
Income	0.014	0.842	−0.001	.992	−0.004	0.940
Distance	0.005	0.947	0.023	0.740	−0.047	0.397
Independent travel					0.294	**<0.001**
Social convenience					0.524	**<0.001**
Safety/Security					0.068	0.248
**DIRECT EFFECTS**						
Child age	−0.222	**0.002**	0.031	0.644	0.096	0.059
Child gender	0.032	0.615	0.001	0.988	0.083	0.085
Parent gender	−0.007	0.911	−0.071	0.276	−0.029	0.565
Education	−0.198	**0.002**	−0.047	0.492	0.009	0.874
Income	0.014	0.842	−0.001	0.992	−0.005	0.911
Distance	0.005	0.947	0.023	0.740	−0.060	0.179
Independent travel					0.294	**<0.001**
Social convenience					0.524	**<0.001**
Safety/Security					0.068	0.248
**INDIRECT EFFECTS**						
Child age					0.001	0.981
Child gender					0.003	0.939
Education					−0.038	0.347
Income					0.001	0.988
Distance					0.012	0.749

Bold values indicate p < 0.05.

In summary, social convenience was directly related to car choice, as was independent travel. Sociodemographics were directly related to safety/security, but distance did not impact on either the stated reasons or mode choice.

## Discussion

This study explores parental travel mode choice based on the assumption that other factors than sociodemographic and environmental ones underlie the mode decision. Previous attempts at understanding parental mode choice have often centered on what certain factors that explain travel mode choice, while our work aims to broaden this knowledge. Given that parents' perceptions of the way their children travel to school are likely to impact upon mode choice, we additionally address what affects these stated reasons and how they then relates to travel mode choice.

Our study shows that parents' stated reasons can be described as Social convenience (relating to these parents' wishes to accompany their children and with the convenience of car) and Safety/security (relating to these parents' concerns about their children encountering difficulties on their way to school). However, analyses show that only Social convenience influences the actual car choice. Safety concerns are a recurrent finding during research into parental travel mode choice; the reason why this finding did not emerge in this study may be the result of the children in this study being aged between 10 and 15, and generally more capable in traffic than younger children. Indeed, the mean value of safety/security was relatively low (*M* = 2.21) supporting this theory. The initial analyses did show a correlation between safety/security and car yet when other factors were accounted for this effect disappears. Moreover, Swedish children become autonomous at a younger age than children do in many other countries (Johansson, 2006), thus it is likely that our results are more comparable to studies of slightly older children elsewhere. Also, many of the studies into children's mode choice, where safety issues are prominent findings, emanates from the US where fear of child abduction is greater than in the Nordic countries where this phenomenon is rather rare (Shutt et al., 2007). Since so many earlier studies have found safety to be a strong incentive for car use, this result may reveal some degree of hopefulness in the otherwise so negative trend in car travel; if security does not account for the increase in children's car travel (in Sweden) it may leave some room for travel change. To reduce parents' worry for their children's safety would require long-term multilevel interventions and changes (e.g., safer roads, traffic lights, pavement shoulders—but also try to change their perceptions of security; Lee et al., 2013).

Social convenience did directly influence mode choice. Parents seem to base their decision to use the car on the fact that it brings them time traveling together with their children. This may additionally be one of the few times during the day when parents get the chance to be alone with their children making plans for the day (Barker, 2009). In this study, Social convenience is based upon *conveniently going that way* and *accompaniment by a parent/adult* and it may be challenging to get parents to choose alternative modes for school journeys since convenience has previously been strongly associated with car choice (Larsen et al., 2015). Parents who believe car is the most convenient and quick way of travel may be reluctant to change to alternative travel modes, since they appear to be more time consuming and thus negatively affect the hectic morning routines. Parents who appreciate the car for its convenience are less likely to let their child actively travel to school (McMillan, 2007). Parental time constraints is a factor that needs to be taken into consideration by policymakers if there is a hope to increase rates of active school travel (McDonald, 2008). Flexible school start may for instance be a means of meeting diverse needs and time constraints of families.

The results further show that distance does not affect the decision to take the car, which supports some research suggesting that parents drive their children regardless of proximity (Ermagun and Levinson, 2016; Woldeamanuel, 2016). A long distance is a major barrier to active travel (Mitra, 2013); however, contradictorily, a short distance does not make parents stop using the car. If parents drive their children even though distance allows active and/or independent travel, there seem to be additional reasons for car use and our finding challenges the common belief that a main reason for taking the car is a long distance.

Sociodemographic factors also affect the reasons stated. Parents with older children valued safety/security less when deciding to take the car. Older children are more capable in traffic and parents' concerns over stranger danger are likely to be more prominent when the mode decision concerns younger children. Parents' educational levels had a direct impact on safety/security whereby parents with a higher level of education indicate to a lesser degree that safety/security impacts upon their decision to take the car. We can only speculate as to why these results have emerged, but the literature has shown that education lessens the perception of riskiness in certain areas (Knight et al., 2003). Perhaps a higher level of education provides skills for calculating the potential danger, or maybe education changes attitudes to security and safety. Education may also affect how we value news and media reports—i.e., that these do not always mirror reality, but address our fears instead. However, one study found that higher degree of education (amongst mothers) increased safety concerns and car use (Mehdizadeh et al., 2016). Our distinct findings would benefit from further investigation in trying to disentangle how sociodemographic factors relates to safety/security. There seem to be different perceptions of safety for different groups and any program aimed to increase security may fail without a solid understanding of how these groups differ in this aspect.

Finally, and quite naturally, if the child was not allowed to travel independently, the parents indicated they chose the car more frequently for school journeys. In this paper, a “no” answer may still indicate that the child is allowed to travel on the school bus or using an active mode together with friends/siblings. In future studies, it would be valuable to additionally investigate what factors affect the “either/or decision” as regards allowing or not allowing the child to travel independently to find alternative solutions to car travel. For instance, the arrangement of organized active travel in groups could be an alternative to car travels.

With our empirical findings, we verify earlier research into children's travel, but we also present evidence regarding how parents' stated reasons impact upon their decision to take the car. These insights bring us one step closer to realizing the full range of factors affecting parents' travel mode choices. With earlier research showing that children enjoy and experience greater satisfaction with the active mode and the school bus (Westman et al., 2016), parents should be made aware that they are instead doing their children a disservice by choosing the car. Not only is the car an unsustainable travel mode, it also deprives children of opportunities for physical activity and of the enjoyment of being with friends on the school bus (Jones et al., 2012). In cases where independent travel is unrealizable, parents could take the opportunity to accompany their children by actively traveling to school and thus fulfilling the desire for social convenience. Some implication of these findings is that policymakers and advocates wishing to decrease children's car travel to school must also consider how to address the convenience issue with car and parental time constraints. Current policies aimed to increase active commuting usually try to do so by increasing safety along school roads. Perhaps this is not the most efficient way to make parents trade in car for active travel since safety/security is not affecting car choice; it is rather a question of social convenience. Programs that address both safety issues but also with the intention to relieve parents' need to escort the child may have better potential to reduce car travel. Such a program is Walking School Buses where parents take turn in walking groups of children to school. By such, the child arrives to school safely on time (McDonald, 2008) and parents get to fulfill the need for accompanying the child to school.

## Ethics statement

This research does not fall under the Act on the Ethical Review of Research Involving Humans (Swedish Code of Statutes 2003:460), according to the Swedish National Research Ethics Committee at Uppsala University. The research was conducted in accordance with approved research protocols. The procedure ensured that the participants were informed that their confidentiality would be maintained.

## Author contributions

All authors listed have made a substantial, direct and intellectual contribution to the work, and approved it for publication.

### Conflict of interest statement

The authors declare that the research was conducted in the absence of any commercial or financial relationships that could be construed as a potential conflict of interest.

## Footnotes

^1^In order to further assess the robustness of the findings we conducted a PLS-MGA (multi group analyses) in which we tested if the results differed between those allowing their children to travel alone and those not allowing it (independent travel; yes/no). We thus removed the independent travel variable from the model and used it as a group comparison variable. The results yielded similar model estimates, giving support to the importance of social convenience regardless of independence or not.
